# The Medical Mind

**Published:** 1909-09-25

**Authors:** 


### The Medical Mind.

One of the most philosophic of politicians confided lately that whereas he could support praise, or blame, with a reasonable amount of the fortitude proper to each case, yet he always felt nervous when he heard himself explained. The public is naturally enough always interested in an "explanation" of the medical profession—since its work concerns everyone so nearly—and therefore plenty of writers arise to supply the demand. Sir Arthur Conan Doyle "explained" in *Round the Red Lamp*, and in spite of the personal note sounded in the title, *Confessio Medici* owed some part of the interest it aroused to the same cause. *Round the Red Lamp* found a large audience; *Confessio Medici* (with a growing amount of subsequent work of identical authorship) a choice one: many medical men, then, will examine the latter with some trepidation, looking to see whether a certain fastidious conception of how they should be represented is realised. Not a few, perhaps, will be disappointed, will sympathise with the politician quoted.

One must not, of course, deny to the literary artist the right to choose his material; and, on the other hand, it is unfair to expect original genius for medicine to be accompanied by original genius for literary expression. Sir Thomas Browne's rare fancies will last as long as the language; but he could not finish anatomising a stranded whale because it smelt badly. John Hunter—inarticulate by comparison—would, it is entirely warrantable to suppose, have had a stronger stomach. Rich and rare as is the mental endowment of the anonymous author of *Confessio Medici*, to a certain class of professional critics he seems lacking in one important quality. His mind is not scientific, or, at least, it is not scientific enough to give laymen just ideas on medical affairs. One passage alone demonstrates this to the cold critic in question. Something in modern psychology revolts the writer's human sympathy, and he conveys admirably his great delight over something later still which upsets the first conclusion. Now, for the truth-seeker, the lover of truth at all costs, human sympathy would never come into the question at all. He would be glad of a result which went to confirm a certain theory, but, even so, with the sobering recollection that he must guard against subconscious, or even conscious, mental bias. To him any fact in connection with a subject of study is *per se* a thing of intense potential interest, a bit of equipment for the game he loves so well—the game, that is, of putting things together, making something out of them, and then seeing if he is right after all. All the lyric touches in *Confessio Medici*, all the passages where clearly the mind is "red-hot behind the pen," concern matters of almost general human interest—early struggles, the growth of income, the four years of extra life given to an old woman. When the author touches, rarely enough, the scientific triumphs of medicine, he drops into what our hard, chilly critic would style, in the words of Rudyard Kipling, "ordinary journalistic prose." He mistrusts individual aspiration in the pursuit of culture beyond that involved in a busy clinician's life, and the more scrambling and competitive this is the more it seems to do his heart good to contemplate it. Rigid orthodoxy and ecclesiastical friendships are other ideals indicated. He hates Christian Science, not because it is erroneous, but because it does harm.

All this may be humane and practical enough, remarks our critic, developing his theme; but it is not the essential spirit of medicine, which is derived from the combative, questioning spirit of science. What has in all ages animated the elect of the medical profession, we hear him continue relentlessly, has been love of knowledge, not concern for humanity. Through prejudice or mistaken tact, biographers are misleading on this point; and yet it is recorded that Helmholtz, complimented on his benefactions to mankind, frankly replied that he merely set out to answer to his own satisfaction certain questions arising in his mind. Fiction, however, from this point of view, is often more truthful. Chaucer's Leech, Scott's Henbane Dwining (less the carefully infused raw-head-and-bloody-bones element), Balzac's Despleins—these pictures are not so taking to the ordinary layman as is *Confessio Medici*, but they represent much better to the cold, clear scientific eyes of our critic what he believes to be the essential medical mind.

